# Force-Bioreactor for Assessing Pharmacological Therapies for Mechanobiological Targets

**DOI:** 10.3389/fbioe.2022.907611

**Published:** 2022-07-19

**Authors:** Austin J. Scholp, Jordan Jensen, Sathivel Chinnathambi, Keerthi Atluri, Alyssa Mendenhall, Timothy Fowler, Aliasger K. Salem, James A. Martin, Edward A. Sander

**Affiliations:** ^1^ Roy J. Carver Department of Biomedical Engineering, College of Engineering, University of Iowa, Iowa City, IA, United States; ^2^ Division of Pharmaceutics and Translational Therapeutics, College of Pharmacy, University of Iowa, Iowa City, IA, United States; ^3^ Department of Orthopedics and Rehabilitation, Carver College of Medicine, University of Iowa, Iowa City, IA, United States

**Keywords:** blebbistatin, collagen, fibrin, fibroblasts, fibrosis

## Abstract

Tissue fibrosis is a major health issue that impacts millions of people and is costly to treat. However, few effective anti-fibrotic treatments are available. Due to their central role in fibrotic tissue deposition, fibroblasts and myofibroblasts are the target of many therapeutic strategies centered primarily on either inducing apoptosis or blocking mechanical or biochemical stimulation that leads to excessive collagen production. Part of the development of these drugs for clinical use involves *in vitro* prescreening. 2D screens, however, are not ideal for discovering mechanobiologically significant compounds that impact functions like force generation and other cell activities related to tissue remodeling that are highly dependent on the conditions of the microenvironment. Thus, higher fidelity models are needed to better simulate *in vivo* conditions and relate drug activity to quantifiable functional outcomes. To provide guidance on effective drug dosing strategies for mechanoresponsive drugs, we describe a custom force-bioreactor that uses a fibroblast-seeded fibrin gels as a relatively simple mimic of the provisional matrix of a healing wound. As cells generate traction forces, the volume of the gel reduces, and a calibrated and embedded Nitinol wire deflects in proportion to the generated forces over the course of 6 days while overhead images of the gel are acquired hourly. This system is a useful *in vitro* tool for quantifying myofibroblast dose-dependent responses to candidate biomolecules, such as blebbistatin. Administration of 50 μM blebbistatin reliably reduced fibroblast force generation approximately 40% and lasted at least 40 h, which in turn resulted in qualitatively less collagen production as determined via fluorescent labeling of collagen.

## Introduction

Tissue fibrosis is a major health issue that impacts millions of people and is costly to treat. It is estimated that fibrotic disorders contribute to 45% of deaths in the United States (Wynn, 2004). For example, idiopathic pulmonary fibrosis alone has a mortality rate of approximately 19 in every 100,000 people (Dove et al., 2019), with an estimated annual cost of about $20,000 per patient (Diamantopoulos et al., 2018). Increased tissue stiffness is also associated with cancer (Barton et al., 1999) and cardiovascular disease (Sidney et al., 2016), the top two leading causes of death in the United States (Lampi & Reinhart-King, 2018). Fibrosis can also occur following trauma to diarthrodial joints, such as the knee or elbow, which can lead to a reduced range of motion (Evans et al., 2009). Between 3% and 10% of patients who undergo total knee arthroplasty (Abdul et al., 2015) and about 8% of patients that receive surgical treatment of the elbow for trauma develop arthrofibrosis (Wessel et al., 2019). Despite the high prevalence of fibrosis and a broad understanding of injury-induced fibrogenesis, few effective anti-fibrotic treatments are available.

**FIGURE 1:**
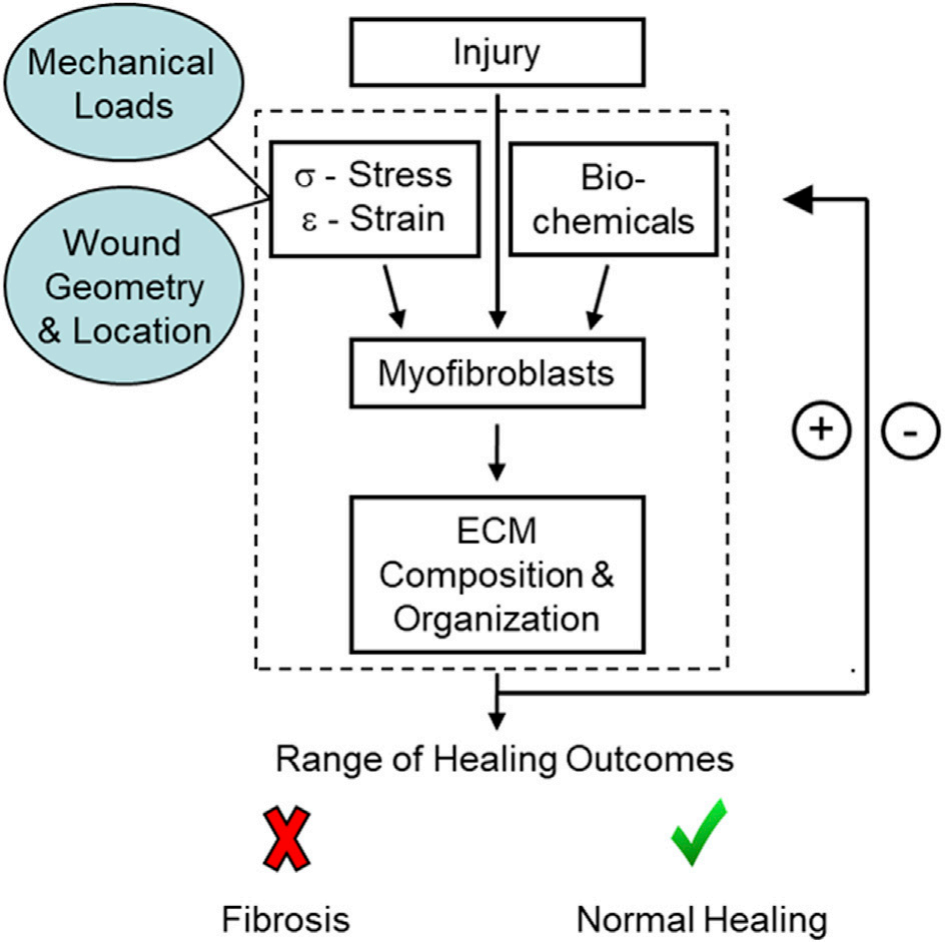
Schematic of the mechano-chemical feedback loop present during healing. Initial conditions at the site of injury, such as the location, mechanical loads, and wound geometry, can affect myofibroblast differentiation and, in turn, healing outcomes.

A common feature of fibrotic tissue is the chronic presence of activated myofibroblasts (Hinz et al., 2007, Hinz et al., 2012; Wynn, 2008; Klingberg et al., 2013). Myofibroblasts are a fibroblast phenotype often distinguished by the expression of alpha smooth muscle actin (α-SMA) in the cytoskeleton and their ability to generate large traction forces and deposit extracellular matrix (ECM) proteins such as collagen (Unterhauser et al., 2004). These cells are also highly responsive to their mechanical environment and the presence of biochemical factors, such as transforming growth factor beta (TGF-β1). Changes in the local mechanical environment are sensed primarily through focal adhesions and the auxiliary proteins that connect the actin cytoskeleton to the surrounding ECM (Geiger et al., 2009). These proteins can activate multiple signaling pathways, such as Rho/ROCK (Geiger & Bershadsky, 2002), Hippo (Ibar et al., 2018), ERK (Matsumoto et al., 2011), and YAP/TAZ (Dupont et al., 2011), which facilitate and reinforce actomyosin contractility, the transmission of force between the cell and the ECM, and the increased synthesis and deposition of collagen and other ECM proteins that further stiffen the tissue locally (Bolós et al., 2010; Guan, 2010). These processes, combined with biochemical signaling, stimulate and enhance a dynamic mechano-chemical feedback loop that can either resolve normally or lead to tissue dysfunction and disease (Figure 1).

Due to their central role in fibrotic tissue deposition, fibroblasts, and myofibroblasts have been the target of many therapeutic strategies centered primarily on either inducing apoptosis or blocking mechanical or biochemical stimulation. For example, Pirfenidone and Nintedanib are small-molecule drugs approved by the FDA for use in treating idiopathic lung fibrosis (Selvaggio & Noble, 2016). Pirfenidone acts by inhibiting TGF-β1 signaling and Nintedanib acts by inhibiting tyrosine kinase receptors for vascular endothelial growth factor (VEGF), fibroblast growth factor (FGF), and platelet-derived growth factor (PDGF) (Lampi & Reinhart-King, 2018). Rapamycin and sirolimus are also FDA-approved drugs that inhibit the kinase mTOR. Rapamycin also hinders macrophage and myofibroblast activation and TGF-β1 release in chronic kidney disease (CKD) (G. Chen et al., 2012; Tulek et al., 2011). The anti-inflammatory drug, sulfasalazine, promotes myofibroblast apoptosis by inhibiting κβ kinase and has been shown to reduce joint stiffness in a leporine model of arthrofibrosis (Atluri et al., 2020a). Additionally, small-molecule inhibition of focal adhesion kinase (FAK) can reduce scar formation in an *in vivo* murine model, thus indicating the potential for small-molecules to block mechanical signaling and reduce fibrosis at the level of the focal adhesion (Wong et al., 2011).

Part of the development of these drugs for clinical use involves *in vitro* prescreening. Typically, prescreening is done using simple high throughput assays on a cell monolayer (Astashkina et al., 2012). Although these assays can yield valuable information, such as drug effects on cell viability, proliferation, and metabolic function, the 2D testing environment is different from the 3D *in vivo* microenvironment, which can substantially impact cell behavior, function, and drug responsiveness (Fernandes & Vanbever, 2009). Crucially, these 2D screens are not ideal for discovering mechanobiologically significant compounds that impact functions like force generation, as force generation and other cell activities are highly dependent on the conditions of the microenvironment. Thus, higher fidelity models are needed to better simulate *in vivo* conditions and relate drug activity to quantifiable functional outcomes.

3D hydrogels can improve drug screening because they better mimic aspects of the tissue’s mechanical and compositional environment. Collagen and fibrin gels are popular choices in this regard because collagen is the most abundant protein in soft tissue matrix and fibrin is the primary structural component of the provisional matrix of a clot (Tuan et al., 1996; Grinnell, 2000). For example, (Saito et al., 2012; Mastikhina et al., 2020) used collagen gels to test the effects of Pirfenidone on gel contraction. They found that Pirfenidone significantly reduced gel contraction in a dose-dependent manner. Along similar lines, Asmani et al. (2019) used a custom micropillar membrane-stretching system to examine how Nintedanib and Pirfenidone affect the response of lung fibroblasts under cyclic strain. Both drugs were shown to decrease forces generated by the fibroblasts.

We also have been investigating the therapeutic potential of different compounds to control the mechanobiology of myofibroblast activity, specifically in the context of fibrosis and contracture in injured joints (Atluri et al., 2016). In particular, we have been exploring the potential of blebbistatin for treating joint capsule fibrosis (Atluri et al., 2020b). Blebbistatin is a small, membrane-permeable biomolecule that reversibly inhibits non-muscle myosin II (NMMII) in a dose-dependent manner (Cheung et al., 2001; Straight et al., 2003; Kovács et al., 2004; Limouze et al., 2004; Allingham et al., 2005; Eddinger et al., 2007; Farman et al., 2008). This molecule limits the ability of the myosin head to discharge bound ADP, which prevents continuation of the power stroke and the generation of traction forces through the actin cytoskeleton. This process also interferes with mechanosensing and has the potential to interrupt the proposed mechanochemical feedback loop that contributes to tissue fibrosis (Atluri et al., 2020b). To provide some guidance on drug dosing strategies for blebbistatin and other drugs in our animal models, we are using fibroblast-seeded fibrin gels as a relatively simple mimic of the provisional matrix of a healing wound. These gels are maintained within a custom-built force-bioreactor that allows us to measure relationships between drug concentration, drug carrier composition, force generation, and downstream collagen production. In the bioreactor, fibroblasts are suspended homogeneously within a fibrin gel. The cells generate traction forces that reduce gel volume and deflect an embedded cantilever wire in proportion to the force generated. The force-bioreactor is coupled with a microcontroller-based imaging system that enables hourly imaging of multiple samples over extended periods of time. This system is a useful *in vitro* tool for quantifying myofibroblast dose-dependent responses to candidate biomolecules.

## Materials and Methods

### Cell Culture

Rabbit joint capsule fibroblasts (RJCFs), obtained from ATCC (HIG-82/CRL-1832), were cultured in Dulbecco’s modified Eagle medium (DMEM; Life Technologies Corporation, Grand Island, NY), supplemented with 10% fetal bovine serum (FBS; Life Technologies Corporation, Grand Island, NY), 0.1% amphotericin B (Life Technologies Corporation, Grand Island, NY) and 1% penicillin/streptomycin (Life Technologies Corporation, Grand Island, NY) in a humidified incubator at 37°C and 5% CO_2_.

### Force-Bioreactor

**FIGURE 2:**
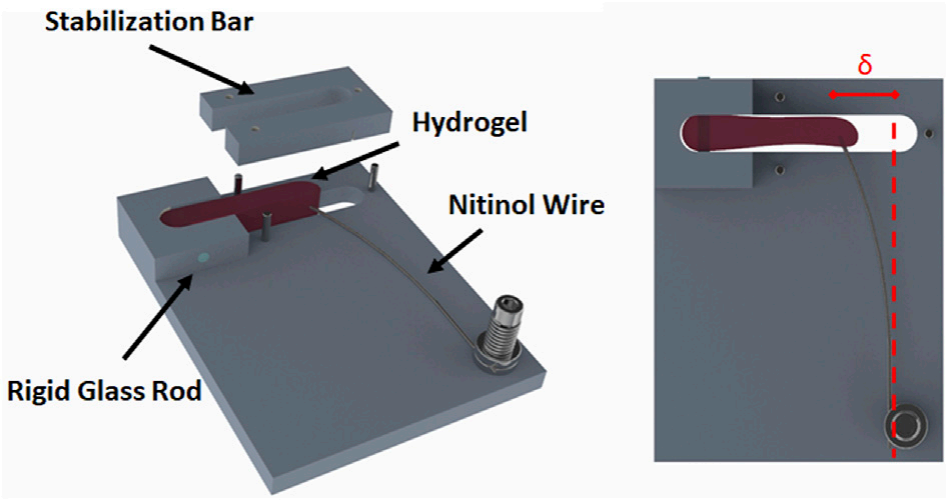
Schematic of the force-bioreactor. A cell seeded fibrin gel (i.e., hydrogel) is polymerized in the cavity containing a rigid glass rod and a bendable Nitinol wire. The stabilization bar is only present during initial gel polymerization. Once polymerized, the stabilization bar is removed, and the hydrogel is free to contract. Force is determined by the amount of deflection (δ) the contracted gel produces in a calibrated wire.

Six force-bioreactors (Figure 2) were machined from 3/8″ Teflon bar stock on a Haas TM-1 CNC mill. The bioreactor body has a 1–5/8″ by 1/4″ by 3/8″ rectangular cavity with a rigid 1.5 mm diameter glass rod and a bendable 0.008″ diameter Nitinol wire (Cat. # WSE000800000SG, Confluent Medical Technologies, Scottsdale, AZ) on either end. The base of a Nitinol (NiTi) wire was clamped to the device between two stainless steel washers and a 0.500″ long, 0.18″ OD, 0.144″ ID corrosion-resistant compression spring (Cat. # 9002T15, McMaster-Carr, Elmhurst, IL). The wire, spring, and washers were compressed with a 4–40 × 1/2″ stainless steel screw that threads into the Teflon base. The glass rod serves as a rigid constraint embedded within the polymerized fibrin gel and the wire acts as a cantilever. The top segment of the bioreactor, also referred to as the stabilization bar, is used to control the casting volume of the fibrin gel until it polymerizes. This was mated to the body with three 1/16″ stainless steel dowel pins. An additional 10 mm thick piece of polysulfone was machined to the diameter of the dish with a cutout for the bioreactor to reduce the volume of medium required to cover the gel. A schematic and parts list for the bioreactor can be found in the Supplementary Materials.

### Nitinol Wire Calibration

**FIGURE 3:**
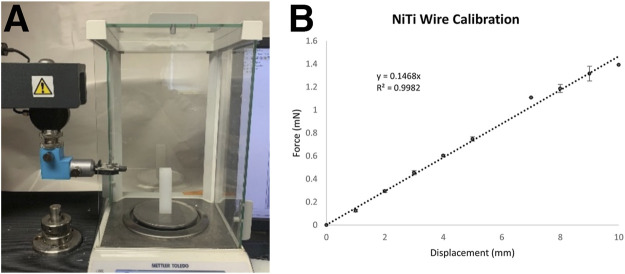
**(A)** The tensile testing system and analytical balance used to generate force-deflection curves for each Nitinol wire. **(B)** A calibration curve generated from the average response of three Nitinol wires.

Each NiTi wire was gripped at its base in a tensile testing machine (TMI Model 84-01) and incrementally displaced downward (Figure 3). The free end of the wire deflected against a Teflon block positioned on an analytical balance (Mettler-Toledo, Columbus, OH). The length of the wire between the grip and the block was set equal to the length of the wire used for the device. At each increment of displacement, the mass displayed on the balance was recorded and converted to force. These data were used to create a calibration curve from linear regression of the measurements from three wires. The resulting equation is F=0.1468 mN/mm×δ , where F is the force in mN and δ is the deflection in mm.

### Cantilever Bioreactor Sterilization

The bioreactor components were placed in a 2% Alconox solution (Alconox, White Plains, NY) and sonicated for 1 h at 60°C. The components were thoroughly rinsed in deionized water and submerged in 70% ethanol overnight. Each piece was placed on a sterile drape within a biosafety cabinet and exposed to UV light for 30 min per side. The Teflon components and a disposable tissue culture dish (Falcon, 100 × 20 mm) were then soaked in a 2% Pluronic F-127 solution (Sigma, St. Louis, MO) for 2 h and allowed to dry for another 2 h. The device was then assembled under sterile conditions and was sealed to the dish using a sterile silicone-based grease (Dow Corning High Vacuum Silicon Grease).

### Fibrin Gel Polymerization

RJCFs were embedded in fibrin gels at a cell density of 1 million cells/ml. Gels were produced as described previously (Sander et al., 2011; El-Hattab et al., 2020) by combining cells with dissolved fibrinogen (F-8630, Sigma, St. Louis, MO), 20 mM HEPES buffered saline (H-0887, Sigma, St. Louis, MO), calcium chloride (Avantor, Center Valley, PA), and dissolved thrombin (T-4648, Sigma, St. Louis, MO). 2 ml of this solution were then added to the cavities of each bioreactor and allowed to polymerize at room temperature for 10 min before being transferred to the incubator for an additional 30 min of polymerization. After polymerization, the U-shaped stabilization bar was carefully removed, the gel was gently released from the side walls, and 25 ml of DMEM supplemented with 1 ng/ml of TGF-β1(PeproTech, Inc., Cranbury, NJ) and 50 μg/ml of ascorbic acid was added to the culture dishes. The bioreactors were maintained for up to 6 days. No media changes were made to prevent disruption of the gel-wire configuration during imaging.

### Imaging and Quantification of Force Production

**FIGURE 4:**
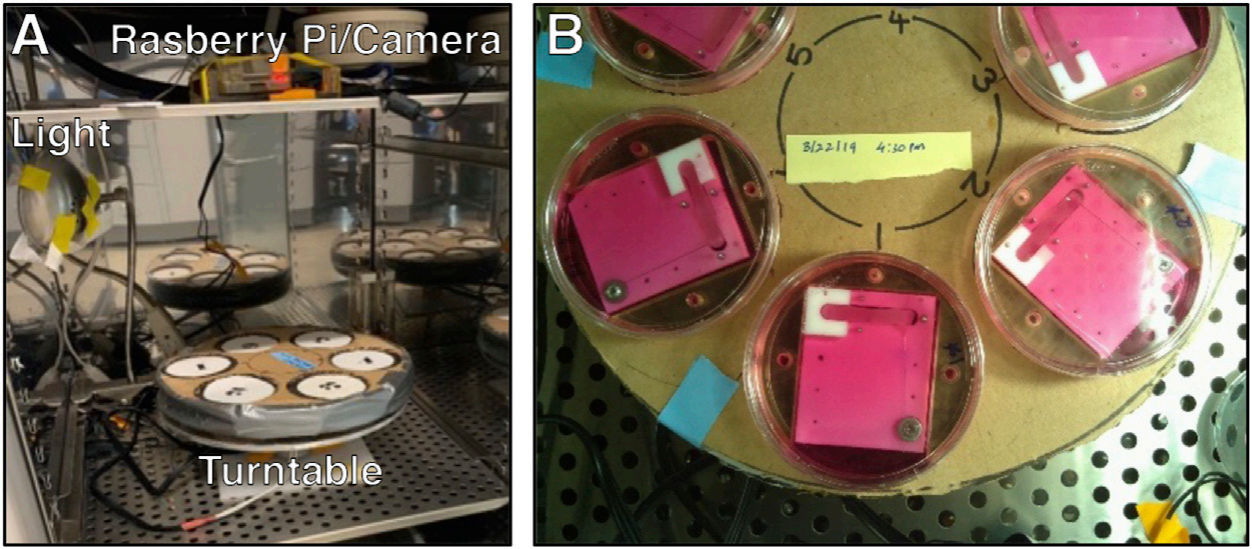
**(A)** The bioreactor turntable and imaging system inside a dedicated incubator. **(B)** View of the turntable from the Raspberry Pi camera.

A Raspberry Pi, camera module (V2: 8 Megapixel, 1080p), and relay-triggered light source were used to acquire overhead images of the bioreactors inside the incubator (Figure 4). Six complete bioreactor assemblies were placed on a custom-built turntable that completed one revolution hourly. Images were acquired every 10 min so that an image of each bioreactor was acquired every hour. After image acquisition, NiTi wire deflection was quantified using a custom MATLAB program and converted to force using the calibration curve. Code for the imaging system can be found in the Supplementary Materials.

### Confocal Imaging of Collagen Content

After the experiment, the gels were removed from the system and placed in a 6-well plate. Cells and collagen were labeled, respectively, using wheat-germ agglutinin conjugated with Alexa Fluor 647 and collagen-binding adhesion protein 35 (CNA35, a kind gift from Magnus Hooke, Texas A&M Health) conjugated with Alexa Fluor 488. Using a Nikon A1 confocal microscope (Nikon Instruments Inc., Melville, NY), representative z-stack images were taken and converted into a 3-D rendering.

### Experimental Conditions

The normal contractile behavior of the myofibroblasts in the system was quantified and compared to the response of the system to the addition of 50 µM blebbistatin (ab120425, Abcam, Waltham, MA) in 0.1% dimethyl sulfoxide (DMSO). Three samples for each condition were tested (*i.e.*, *n* = 3 control and *n* = 3 blebbistatin). Two separate experiments (also with n = 3 per group) were performed that differed in when the drug was added (*i.e.*, 72 or 96 h after the start of the experiment). Thus, the total number of samples was *n* = 12.

### Statistical Analysis

Statistical analysis was carried out using GraphPad Prism version 9.31 (GraphPad, San Diego, CA). Paired t-tests were conducted to assess significance (*p* < 0.05) for differences in force before and after treatment. Unpaired t-tests were conducted to assess significant differences between treatment groups and controls.

## Results and Discussion

**FIGURE 5:**
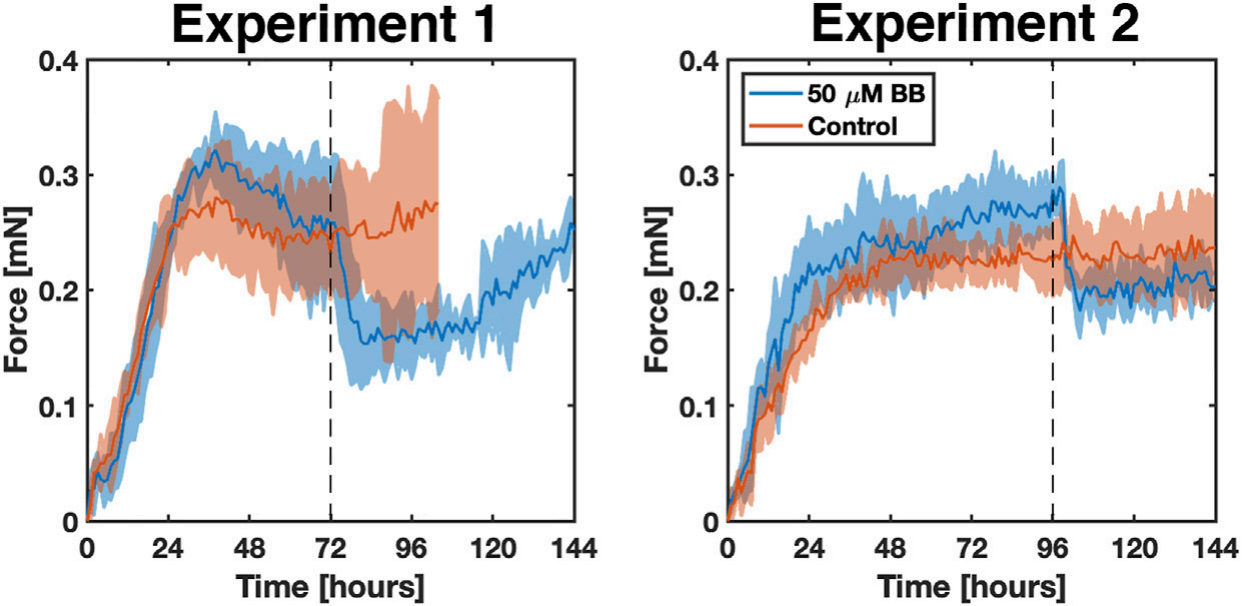
Two experiments comparing the response and reproducibility of the bioreactor systems over 6 days in the presence (blue) and absence (red) of blebbistatin. Solid lines indicate the average of *n* = 3 replicates. The shaded areas correspond to the standard deviation. The grey dotted line marks the time at which 50 μm blebbistatin in 0.1% DMSO was added to the samples.

During 6 days of culture, untreated fibrin gels (*i.e.*, control) rapidly densified and decreased in length and volume from cell-generated traction forces, which were measured from the deflection of the embedded NiTi wires (Figure 5, Supplementary Movie S1). Treated gels followed the same trajectory but relaxed nearly instantly once blebbistatin was administered (Figure 5, Supplementary Movie S2). In the first experiment, the average rate of force increase in both control and treated gels remained nearly constant over the first 26 h at 0.010 mN/h. The control gels then reached an average steady-state force of 0.257 ± 0.012 mN for the remainder of the experiment. Starting around day four, gel compaction and thinning around the wire concentrated the stress such that the gel tore from the embedded wire. The treated gels reached a higher average force of 0.285 ± 0.021 mN between 26 and 72 h. At 72 h, when 50 µM blebbistatin in 0.1% DMSO was added, the average force rapidly decreased 40% from 0.257 ± 0.055 mN to a minimum of 0.154 ± 0.035 mN in 10 h (*p* = 0.0199). After approximately 40 additional hours, the force began to increase again.

**FIGURE 6:**
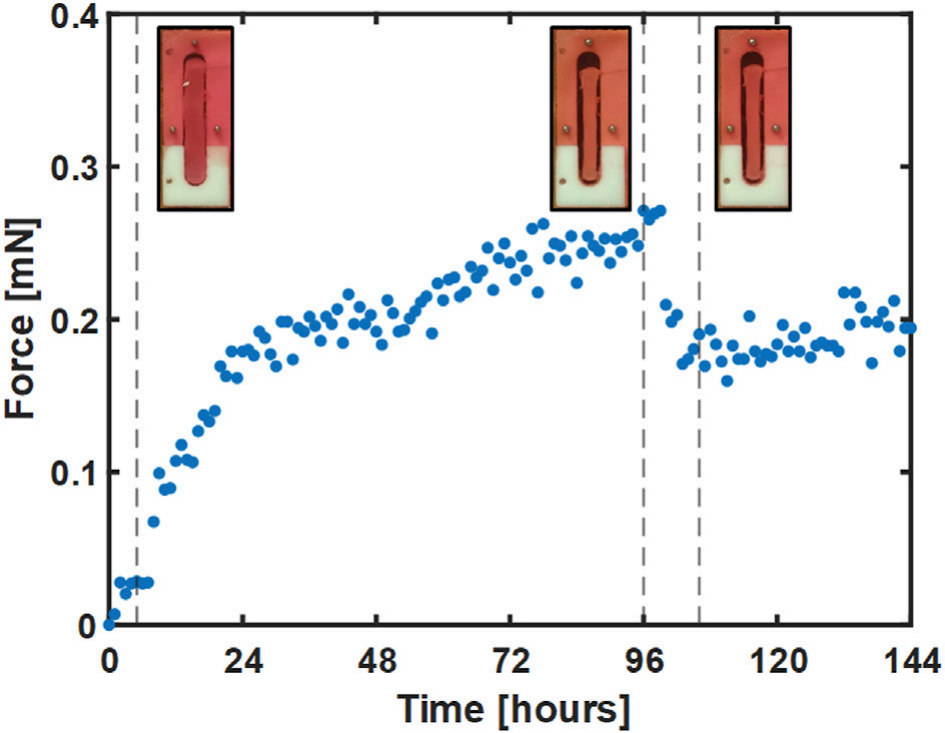
A representative plot of the force response for a single gel treated with blebbistatin at 96 h. Images of gel deflection correspond to 5, 96, and 105 h and are marked with dotted lines.

A second set of experiments was conducted with two changes. First, to help address the issue of gel failure that occurred in the control gels in the first set of experiments, the surface area of each wire was increased by a factor of 8.5 by gluing a short segment (∼5 mm) of a mylar wrapped hematocrit tube (OD ∼ 1.7 mm) to each wire end. In addition, the pre-treatment period was extended by 24 h to help ensure that the gels had reached a steady-state level of tension. Consequently, 50 µM blebbistatin in 0.1% DMSO was added at 96 h to the treatment group. An example gel from this group is shown in Figure 6, where select images of the gel in the bioreactor accompany key parts of the force curve.

Compared to the first experiment, the average rate of force increase was slightly lower over the first 26 h at 0.009 mN/h and 0.007 mN/h for the treated and control gels, respectively. Average forces between 27 and 96 h were also a little lower at 0.249 ± 0.018 mN and 0.220 ± 0.014 mN for treated and control gels, respectively. As before, administration of blebbistatin significantly reduced average gel force 37% from an average peak of 0.289 ± 0.022 mN at 98 h to 0.182 ± 0.024 mN at 104 h (*p* = 0.0389). Average gel force remained low for the remaining 48 h of the experiment, with signs of a slow increase in force beginning to develop near the conclusion of the experiment.

Taken together, our experiments show that administration of 50 µM blebbistatin in 0.1% DMSO reduced cell-generated tension within the gel by approximately 40% with effects that lasted at least 40 h. Small differences between the two independent sets of experiments were not significant and were likely due to the normal biological variation inherent in cell culture experiments.

**FIGURE 7:**
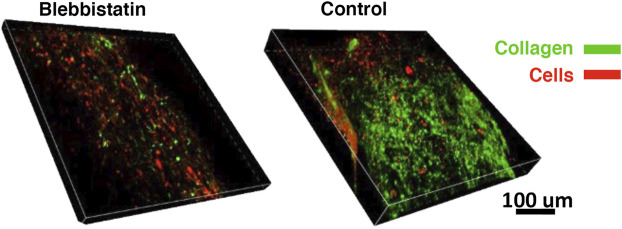
Differences in collagen synthesis visualized on day 6. Collagen deposition (green) and cells (red) depicted in the confocal volume rendering for blebbistatin and control (i.e., no treatment), respectively.

To determine whether the decrease in gel force observed also impacted collagen production, both treated and control gels were also labeled with CNA35 (Figure 7). Qualitatively, less CNA35 labeling was observed in blebbistatin treated gels compared to controls, suggesting that collagen production was lower.

Several groups have used similar techniques to measure cell force generation within hydrogels (Kolodney & Wysolmerski, 1992; Eastwood et al., 1994; Freyman et al., 2001; Campbell et al., 2003). Most directly comparable to the present study, Eastwood *et al.* (Eastwood et al., 1994) measured the forces generated by human dermal fibroblasts in 1 mg/ml collagen gels over 48 h and found a linear increase in force followed by a plateau that started around 24 h. The average peak force per cell was reported as 100 ± 20 pN/cell. Our results using rabbit joint capsule fibroblasts in 6 mg/ml fibrin gels match these findings very closely. In addition to strong parallels in the force curves (*i.e.*, linear increase followed by a plateau around 24 h), our average force per cell ranged between 110 and 145 pN/cell.

It has been suggested that fibroblasts and other cell phenotypes regulate ECM tension according to a cell-specific setpoint in a process termed tensional homeostasis (Brown et al., 1998). At least over short periods of time (*i.e.*, hours to days), fibroblasts appear to regulate the forces they generate irrespective of the material properties of the hydrogel. The fact that we observed comparable force values in fibrin gels compared to collagen gels is supportive of this idea. However, long term adaptation of cells to their mechanical environment suggests that these setpoints can change and may be responsible for a host of fibrotic disorders and other diseases. One can modulate wire stiffness in the bioreactor by changing wire diameter and/or length. Different wire stiffness will impact the balance between cell traction forces and reorganization/realignment of the fibrin fibers in the gel, which might enable one to investigate these force set-points and other mechanobiological processes in more detail.

It is also possible that more subtle changes in gel stiffness from ECM adaptations could be revealed by this bioreactor system, but that requires knowing more about how much of the force remaining after administration of blebbistatin can be attributed to the cells versus forces supported by the gel. The 50 μM concentration of blebbistatin used in this study was chosen because it is not cytotoxic but still effective at reducing cell traction forces and collagen production (Atluri et al., 2016). This concentration, however, does not eliminate traction forces completely. To eliminate traction forces completely, one could try other compounds, such as cytochalasin D (Wakatsuki et al., 2000), or apply techniques that induce apoptosis (Roberts et al., 2004). The remaining deflection in the wire should then correspond to the force supported by ECM adaptations, a phenomenon reported in other hydrogel systems (Wakatsuki et al., 2000; Bidan et al., 2016; Brauer et al., 2019). More work will need to be done to explore this idea further.

This bioreactor was designed to monitor real-time changes in cell traction force in response to drugs that impact the force generating/sensing apparatus of the cell and downstream collagen production. We believe that there are metrics in the force curves that will be predictive of this downstream remodeling process, such as the percent reduction in peak force, the duration of this reduction, and the force magnitudes measured. However, additional experiments and biochemical quantification of collagen content will be necessary to firmly establish these relationships. One could also perform mechanical tests, such as uniaxial extension tests on the gels prior to assaying for collagen, to better relate the resulting collagen content to mechanical properties. This information, combined with the knowledge of how forces distribute among the cells and the gel, will provide additional insight on how the mechanical and biochemical environment influences tissue remodeling and how controlling that environment could be used to improve wound healing and tissue repair.

Our bioreactor system can help us better understand cell and ECM adaptations, particularly in the context of a mechanochemical feedback loop, and how compounds such as blebbistatin can be administered to control the healing response in a tissue. With respect to the latter, a balance must be struck between allowing fibroblasts to synthesize enough collagen and ECM proteins to repair the site of injury and limiting myofibroblast activity so that excessive collagen deposition is prevented. Our next steps are to use this bioreactor with drug delivery systems, such as biodegradable polymer microparticles or liposomes, to determine release kinetics and a therapeutic range of drug concentrations for normal healing.

Tomasek *et al.* (Tomasek et al., 2002) proposed that quiescent fibroblasts differentiate into a contractile, but α-SMA negative, proto-myofibroblast before transitioning to a fully activated α-SMA positive myofibroblast. Generally, it was thought that myofibroblast differentiation was irreversible, but several studies since have indicated that reversal can occur (Kisseleva et al., 2012; Garrison et al., 2013; Kollmannsberger et al., 2018). For example, Kollmannsberger *et al.* showed that myofibroblasts in a 3D microtissue reverted to α-SMA negative fibroblasts once tensile forces in the interior of the growing microtissue were offloaded to newly formed ECM, a process that occurred over a period of 2–3 weeks (Kollmannsberger et al., 2018). An even longer time scale of months was observed for myofibroblast reversal in a model of rat liver fibrosis (Kisseleva et al., 2012). In our experiments, it is highly likely that the duration of blebbistatin exposure was too short for myofibroblast reversal. Future experiments could be extended to examine whether blebbistatin can also cause reversal.

It has been known for decades that fibroblasts have tissue-specific properties that make, for example, the behavior of corneal fibroblasts differs from that of dermal fibroblasts (Doane & Birk, 1991). It is now clear that even within a specific tissue there is quite a bit of fibroblast heterogeneity. For example, genetic lineage tracing experiments in mice have found at least two distinct fibroblast populations in the peripapillary and reticular dermis, each with unique functions with respect to tissue repair (Driskell et al., 2013; Rinkevich et al., 2015). Similarly, several genetically and biofunctionally distinct myofibroblast subpopulations have been identified in the healing wounds of mice (Shook et al., 2018, 2020), which suggests that each subpopulation has a unique role in the wound healing process. Heterogeneous tissue-specific fibroblast populations have also been recently identified in other tissues, such as the heart (Tallquist, 2020) and the lung (Xie et al., 2018). In this study, we used a relevant tissue-specific fibroblast population from the joint capsule in order to simplify our understanding of the relationships between drug dosing and joint capsule fibroblast behavior. As we develop this system further, it will be necessary to consider the effects and interactions of a more heterogenous cell population, particularly in the instance of a contracted joint, where additional cells, particularly immune cells, migrate into the capsule and impact the healing response (Qu et al., 2019). Building this additional complexity into our system has the potential to enable a deeper understanding of how joint contracture develops and how various treatment strategies might be developed in conjunction with animal studies.

**FIGURE 8:**
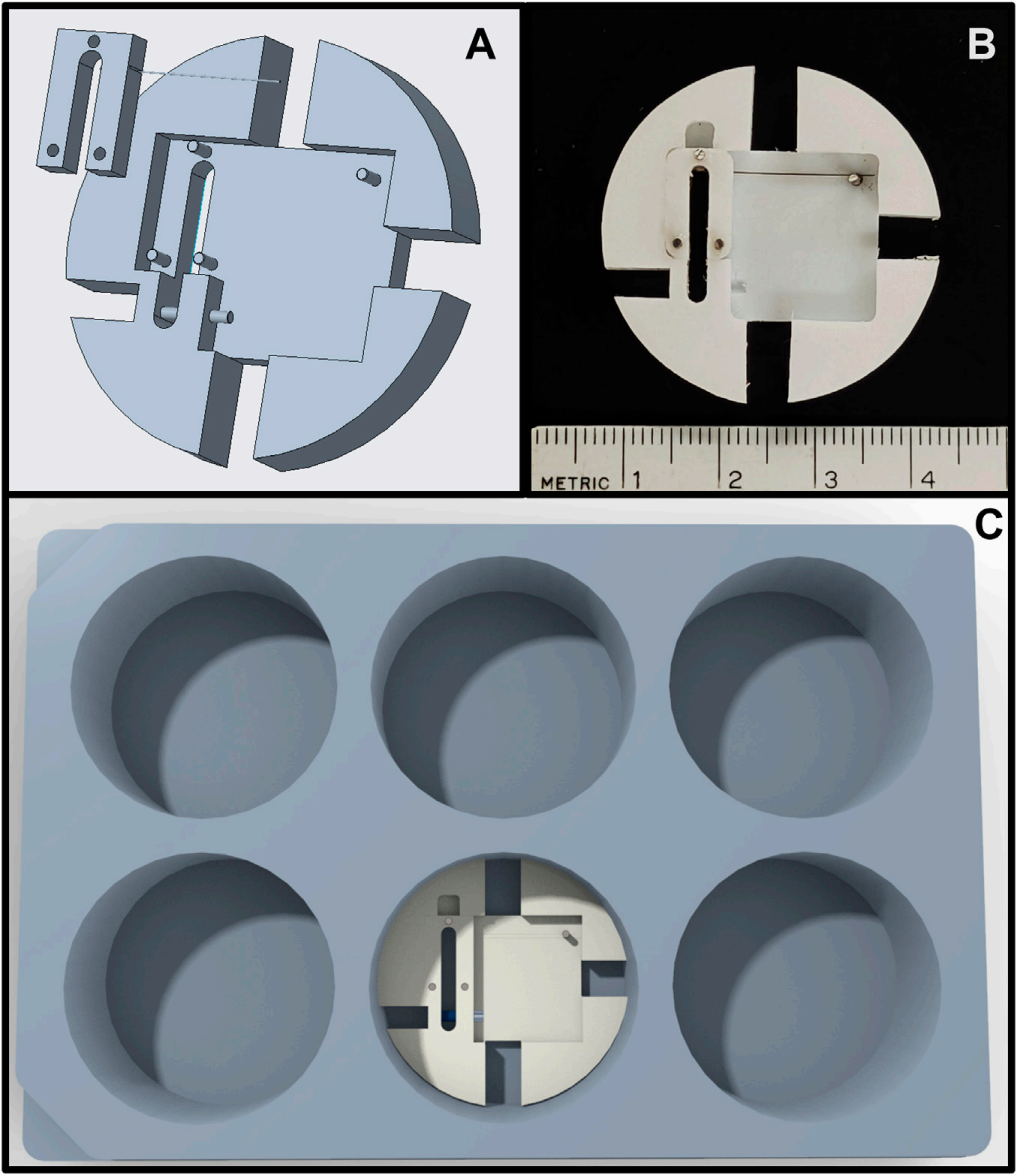
**(A)** Rendering of the scaled-down force-bioreactor. **(B)** Image of a prototype bioreactor. **(C)** Rendering of the scaled-down bioreactor inside a standard 6-well tissue culture plate.

The bioreactor sample size is well-suited for biochemical ECM quantification, mechanical testing, and for having sufficient cell numbers for conventional RNA or DNA analysis (*e.g.*, RNAseq or RT-qPCR). To accommodate this sample size, however, a large medium reservoir (*i.e.*, 25 ml) is needed to provide a sufficient source and sink for metabolites and waste products. A disadvantage to having a large reservoir is that the amount of drug needed can be costly. To address this issue, we have also designed a scaled-down version of the system that fits inside a 6-well plate (Figure 8) and uses a tenth of the reagents of the current system. Even smaller scale micropillar systems exist that easily fit within a 96-well plate (Vandenburgh et al., 2008; Boudou et al., 2012). These systems are well-suited for high-throughput drug screening and quantifying relationships between various drugs and force generation with minimal usage of reagents. However, the gel sizes generally preclude additional analysis for gene expression, protein quantification, and mechanical testing. Recent developments with single-cell RNA-seq (Luecken & Theis, 2019), new proteomics approaches for quantifying ECM proteins (Jacobson et al., 2020), and nanoindentation/atomic force microscopy (Xu et al., 2016) have the potential to enable one to work with much smaller samples, but these technologies are still being refined and not always available. Therefore, we envision the use of different sized-bioreactors, coupled with computational biochemical and biomechanical models, as the components of a multi-pronged approach to drug-screening for mechanobiologically significant compounds.

These bioreactors could also be used to study the contractile behavior of other mechanobiologically relevant cell phenotypes, particularly those that apply traction forces to their surrounding ECM, such as arterial smooth muscle cells (Chen et al., 1991), retinal epithelial cells (Smith-Thomas et al., 2000), or osteoblasts (Qi et al., 2007). Cells could be studied in the context of other diseases, such as ventilator induced lung injury (Bates & Smith, 2018) or cancer (Northcott et al., 2018), or in the context of development (Nelson & Gleghorn, 2012; Vianello & Lutolf, 2019), or other mechanobiologically relevant areas of investigation.

In conclusion, the force bioreactor system demonstrated that administration of 50 µM blebbistatin reliably reduces fibroblast force generation, which in turn resulted in qualitatively less collagen production as determined via fluorescent labeling. Quantification of collagen content (as well as the synthesis of other ECM proteins) via biochemical or proteomic analysis are needed to confirm these observations and to better understand the implications of temporarily and reversibly interrupting fibroblast/myofibroblast mechanosensing. In addition, tensile testing of the gels would be useful for relating compositional changes to mechanical changes.

## Data Availability

The original contributions presented in the study are included in the article/Supplementary Material, further inquiries can be directed to the corresponding author.
