# Supplementary material for: Force-Bioreactor for Assessing Pharmacological Therapies for Mechanobiological Targets
Source: Front Bioeng Biotechnol. 2022 Jul 19;10:907611. doi: 10.3389/fbioe.2022.907611 (PMC9343955; doi:10.3389/fbioe.2022.907611)
Supplement: Supplementary file 4 [file DataSheet1.docx]

###########################################################################

# Image Collect 2.0

# Author: Austin Scholp, Roy J. Carver of Biomedical Engineering,

% University of Iowa. June 2022.

# Based on code previously written by Alyssa Mendenhall

# Used to collect images periodically from the rotating bioreactor system

###########################################################################

from picamera import PiCamera

from datetime import datetime

from time import sleep

import RPi.GPIO as GPIO

import time

#Initial Setup

relayPin = 12 #Pin number on the Pi board that connects to the power relay

GPIO.setmode(GPIO.BOARD) #Numbers the pins based on board location

GPIO.setup(relayPin, GPIO.OUT) #The relay pin will be an output pin

lightTime = 1.5 #time (in seconds) the program pauses to allow the light to fully turn on

waitTime = 600 #Time (in seconds) between image captures (10 minutes minus the time the light is on)

imagesPerReactor = 24 #the number of images you want to collect per cantilever

camera = PiCamera() #Define camera as the picamera object

camera.resolution = (2592, 1944) # Set the piCamera to the highest resolution

i = 1 #start numbering images at 1

numImages = 0 #at start, no images have been captured

while numImages < (imagesPerReactor*6): #runs until all the desired images are collected. Note, there are 6 reactors

elapsedTime = 0

startTime = time.time()

GPIO.output(relayPin, GPIO.HIGH) #turns on the light

sleep(lightTime) #pauses the program for 2 seconds

now = datetime.now()

currentTime = now.strftime("%Y-%m-%d_%H.%M.%S")

filePath = '/home/pi/Cantilever Bioreactor/Images/'

fileName = "Image_" + currentTime+"_[" + str(i) + "].jpg"

nameANDpath = filePath + fileName

camera.start_preview()

camera.capture(nameANDpath)

camera.stop_preview()

i += 1 #index the image number

# Update i to name images in groups of 6

if i==7:

i=1

#Turn off light

GPIO.output(relayPin, GPIO.LOW)

numImages += 1

message = "Image Captured: " + fileName

print(message)

while elapsedTime < waitTime:

currentTime = time.time()

elapsedTime = currentTime - startTime

print("Image collection complete.")
