# Supplementary figures and images for: Force-Bioreactor for Assessing Pharmacological Therapies for Mechanobiological Targets
Source: Front Bioeng Biotechnol. 2022 Jul 19;10:907611. doi: 10.3389/fbioe.2022.907611 (PMC9343955; doi:10.3389/fbioe.2022.907611)

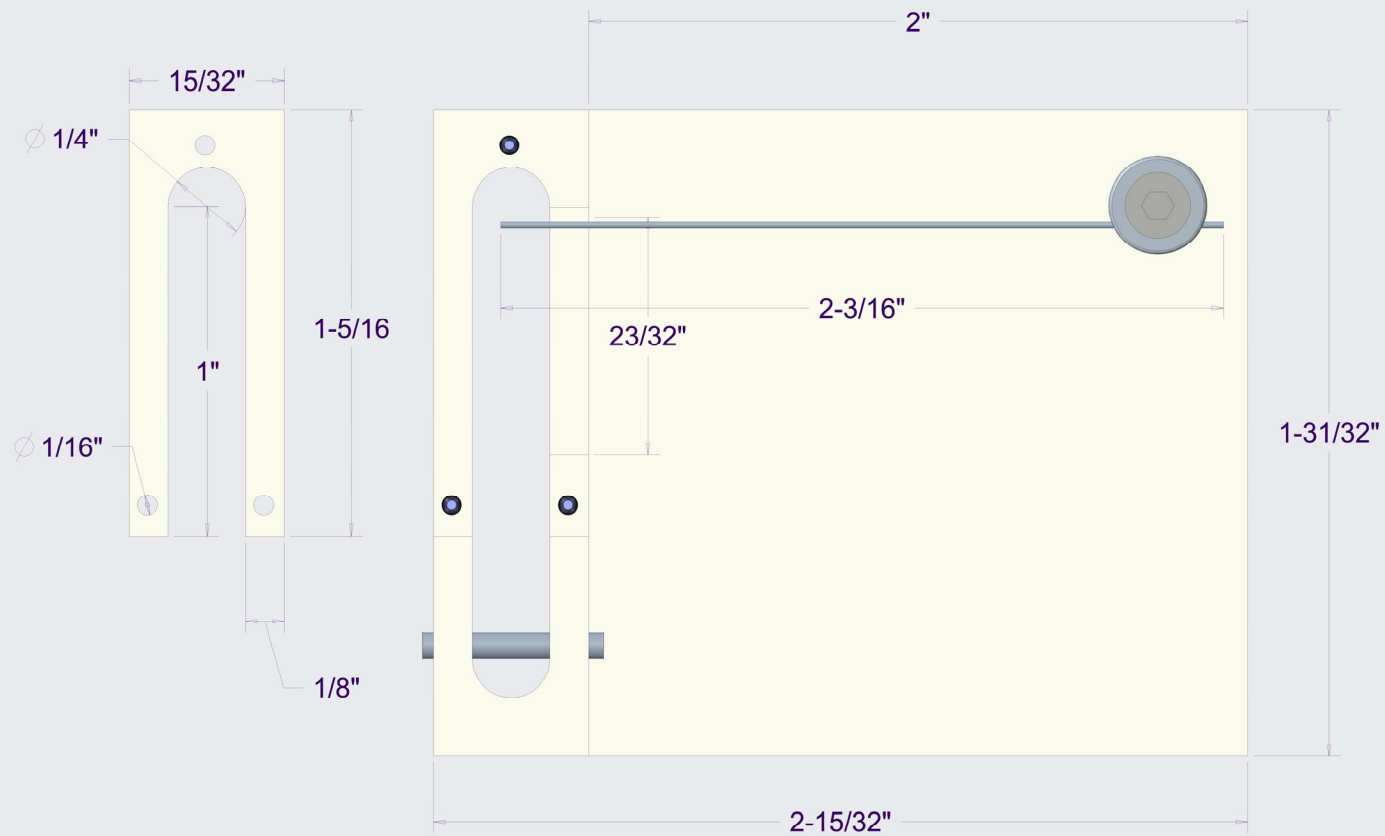

Supplement: Supplementary file 5 [file Image1.pdf]
